# International Analysis of Electronic Health Records of Children and Youth Hospitalized With COVID-19 Infection in 6 Countries

**DOI:** 10.1001/jamanetworkopen.2021.12596

**Published:** 2021-06-11

**Authors:** Florence T. Bourgeois, Alba Gutiérrez-Sacristán, Mark S. Keller, Molei Liu, Chuan Hong, Clara-Lea Bonzel, Amelia L. M. Tan, Bruce J. Aronow, Martin Boeker, John Booth, Jaime Cruz Rojo, Batsal Devkota, Noelia García Barrio, Nils Gehlenborg, Alon Geva, David A. Hanauer, Meghan R. Hutch, Richard W. Issitt, Jeffrey G. Klann, Yuan Luo, Kenneth D. Mandl, Chengsheng Mao, Bertrand Moal, Karyn L. Moshal, Shawn N. Murphy, Antoine Neuraz, Kee Yuan Ngiam, Gilbert S Omenn, Lav P. Patel, Miguel Pedrera Jiménez, Neil J. Sebire, Pablo Serrano Balazote, Arnaud Serret-Larmande, Andrew M. South, Anastasia Spiridou, Deanne M. Taylor, Patric Tippmann, Shyam Visweswaran, Griffin M. Weber, Isaac S. Kohane, Tianxi Cai, Paul Avillach

**Affiliations:** 1 Department of Pediatrics, Harvard Medical School, Boston, Massachusetts; 2 Computational Health Informatics Program, Boston Children’s Hospital, Boston, Massachusetts; 3 Department of Biomedical Informatics, Harvard Medical School, Boston, Massachusetts; 4 Department of Biostatistics, Harvard T.H. Chan School of Public Health, Boston, Massachusetts; 5 Departments of Biomedical Informatics, Pediatrics, Cincinnati Children's Hospital Medical Center, University of Cincinnati, Ohio; 6 Institute of Medical Biometry and Statistics, Faculty of Medicine and Medical Center, University of Freiburg, Germany; 7 Digital Research, Informatics and Virtual Environments (DRIVE), Great Ormond Street Hospital for Children, London, United Kingdom; 8 Department of Health Informatics, Hospital Universitario 12 de Octubre, Madrid, Spain; 9 Department of Biomedical Health Informatics and the Department of Pediatrics, The Children's Hospital of Philadelphia, Philadelphia, Pennsylvania; 10 Department of Anesthesiology, Critical Care, and Pain Medicine, Boston Children’s Hospital, Boston, Massachusetts; 11 Department of Learning Health Sciences, University of Michigan, Ann Arbor; 12 Department of Preventive Medicine, Northwestern University, Evanston, Illinois; 13 Department of Medicine, Massachusetts General Hospital, Boston; 14 IAM Unit, Bordeaux University Hospital, Bordeaux, France; 15 Department of Infectious Diseases, Great Ormond Street Hospital for Children, London, United Kingdom; 16 Department of Neurology, Massachusetts General Hospital, Boston, Massachusetts; 17 Department of Biomedical Informatics, Hôpital Necker-Enfants Malade, Assistance Publique Hôpitaux de Paris, University of Paris, Paris, France; 18 Department of Biomedical informatics, WiSDM, National University Health Systems Singapore, Singapore; 19 Department of Computational Medicine & Bioinformatics, Internal Medicine, Human Genetics, & School of Public Health, University of Michigan, Ann Arbor; 20 Department of Internal Medicine, Division of Medical Informatics, University of Kansas Medical Center, Kansas City; 21 Department of Pediatrics-Section of Nephrology, Brenner Children's Hospital, Wake Forest School of Medicine, Winston Salem, North Carolina; 22 Department of Pediatrics, Perelman Medical School at the University of Pennsylvania, Philadelphia; 23 Department of Biomedical Informatics, University of Pittsburgh, Pittsburgh, Pennsylvania

## Abstract

**Importance:**

Additional sources of pediatric epidemiological and clinical data are needed to efficiently study COVID-19 in children and youth and inform infection prevention and clinical treatment of pediatric patients.

**Objective:**

To describe international hospitalization trends and key epidemiological and clinical features of children and youth with COVID-19.

**Design, Setting, and Participants:**

This retrospective cohort study included pediatric patients hospitalized between February 2 and October 10, 2020. Patient-level electronic health record (EHR) data were collected across 27 hospitals in France, Germany, Spain, Singapore, the UK, and the US. Patients younger than 21 years who tested positive for COVID-19 and were hospitalized at an institution participating in the Consortium for Clinical Characterization of COVID-19 by EHR were included in the study.

**Main Outcomes and Measures:**

Patient characteristics, clinical features, and medication use.

**Results:**

There were 347 males (52%; 95% CI, 48.5-55.3) and 324 females (48%; 95% CI, 44.4-51.3) in this study’s cohort. There was a bimodal age distribution, with the greatest proportion of patients in the 0- to 2-year (199 patients [30%]) and 12- to 17-year (170 patients [25%]) age range. Trends in hospitalizations for 671 children and youth found discrete surges with variable timing across 6 countries. Data from this cohort mirrored national-level pediatric hospitalization trends for most countries with available data, with peaks in hospitalizations during the initial spring surge occurring within 23 days in the national-level and 4CE data. A total of 27 364 laboratory values for 16 laboratory tests were analyzed, with mean values indicating elevations in markers of inflammation (C-reactive protein, 83 mg/L; 95% CI, 53-112 mg/L; ferritin, 417 ng/mL; 95% CI, 228-607 ng/mL; and procalcitonin, 1.45 ng/mL; 95% CI, 0.13-2.77 ng/mL). Abnormalities in coagulation were also evident (D-dimer, 0.78 ug/mL; 95% CI, 0.35-1.21 ug/mL; and fibrinogen, 477 mg/dL; 95% CI, 385-569 mg/dL). Cardiac troponin, when checked (n = 59), was elevated (0.032 ng/mL; 95% CI, 0.000-0.080 ng/mL). Common complications included cardiac arrhythmias (15.0%; 95% CI, 8.1%-21.7%), viral pneumonia (13.3%; 95% CI, 6.5%-20.1%), and respiratory failure (10.5%; 95% CI, 5.8%-15.3%). Few children were treated with COVID-19–directed medications.

**Conclusions and Relevance:**

This study of EHRs of children and youth hospitalized for COVID-19 in 6 countries demonstrated variability in hospitalization trends across countries and identified common complications and laboratory abnormalities in children and youth with COVID-19 infection. Large-scale informatics-based approaches to integrate and analyze data across health care systems complement methods of disease surveillance and advance understanding of epidemiological and clinical features associated with COVID-19 in children and youth.

## Key Points

**Question:**

What are international trends in hospitalizations for children and youth with SARS-CoV-2, and what are the epidemiological and clinical features of these patients?

**Findings:**

This cohort study of 671 children and youth found discrete surges in hospitalizations with variable trends and timing across countries. Common complications included cardiac arrhythmias and viral pneumonia, and laboratory findings included elevations in markers of inflammation and abnormalities of coagulation; few children and youth were treated with medications directed specifically at SARS-CoV-2.

**Meaning:**

These findings suggest large-scale informatics-based approaches used to incorporate electronic health record data across health care systems can provide an efficient source of information to monitor disease activity and define epidemiological and clinical features of pediatric patients hospitalized with SARS-CoV-2 infections.

## Teaser

This cohort study aims to describe international hospitalization trends and key epidemiological and clinical features of children and youth with COVID-19.

## Introduction

The clinical presentation of coronavirus disease 2019 (COVID-19) differs substantially between children and youth and adults. The unique clinical features, complications, and outcomes of COVID-19 among children and youth warrant special consideration in epidemiologic, management, and prevention studies.^1^ However, the low prevalence of disease in children and youth—compounded by the routine challenges of conducting large clinical trials in pediatric populations—has limited their inclusion in many studies.^2^ Key questions remain related to risk factors for severe and rare disease manifestations and optimal use of clinical interventions.^3^ The experience with COVID-19 has highlighted the critical need to have efficient methods to complement traditional clinical investigations and public health surveillance to study pediatric populations during a rapidly evolving pandemic.

Large volumes of clinical data are available in electronic health records (EHRs) to support epidemiological studies of medical conditions and analyze real-world outcomes related to specific populations and interventions.^4^ When used appropriately, these data represent a powerful tool to fill in gaps and address shortcomings of conventional clinical trials. For example, EHR data have been applied to more efficiently assess medication safety in children and youth or to test at scale potential associations between risk factors and pediatric conditions.^5,6^ These data are particularly conducive to the study of small populations or rare events that can be difficult to capture in smaller data sets.^7,8^ Other key benefits of EHR data include the ability to ascertain clinical trajectories and to facilitate multinational studies by combining data across health care systems. Soon, EHR-based observational data may also contribute to assessing the impact of vaccines in children and youth, including efficacy and long-term safety in pediatric subpopulations with limited representation or follow-up in clinical trials.

The Consortium for Clinical Characterization of COVID-19 by EHR (4CE) is an international collaborative covering 351 adult and pediatric hospitals in 7 countries that has collected patient-level EHR data on 39 200 hospitalized patients with polymerase chain reaction (PCR)–confirmed diagnosis of SARS-CoV-2.^9^ The use of common data elements across a federated network allows for integration and harmonization of data to enable analyses of the disease manifestation and epidemiology of COVID-19 across health care sites. Focusing on adult populations, studies have used data from the 4CE initiative to measure the prevalence of specific types of clinical complications, develop EHR-based severity algorithms,^10^ identify laboratory tests predicting severity in patients with COVID-19,^11^ and define country-level differences in demographic and epidemiological presentation.^9^ Leveraging data from this collaborative, our objective was to demonstrate large-scale, multinational use of EHR data to study COVID-19 in children and youth and describe hospitalization trends and key epidemiological and clinical features of the disease.

## Methods

In this cohort study, each participating site obtained institutional review board approval to share deidentified, aggregated patient data with the 4CE consortium. Informed consent was waived because the patient data were deidentified. The study followed the Strengthening the Reporting of Observational Studies in Epidemiology (STROBE) reporting guideline.

### Participating Sites and Cohort Identification

Participating 4CE sites in France, Germany, Spain, Singapore, the UK, and the US reported pediatric-specific data and contributed patients to this cohort analysis. We analyzed patients younger than 21 years who were hospitalized between February 2 to October 10, 2020, and had a positive reverse transcription PCR test for SARS-CoV-2 infection 7 days before to 14 days after the date of admission. Positive tests were identified by local data managers at each site who mapped internal codes for SARS-CoV-2 laboratory results. Demographic information on a subset of patients admitted through April 11, 2020, was previously described.^9^

### Data Extraction and Aggregation

 Several sites included multiple hospitals, and pediatric data were extracted from each hospital participating in the pediatric substudy (eTable in Supplement 1). Certain sites applied obfuscation thresholds to minimize disclosure risks related to small patient numbers. When values were obfuscated, we inserted a value of 0.5 times the obfuscation threshold.

Sites executed queries on local clinical data warehouses containing patient-level EHR data.^9^ To construct the required data files, sites used the Informatics for Integrating Biology and the Bedside (i2b2) platform, the Observational Medical Outcomes Partnership (OMOP) Common Data Model, Epic Clarity, or other clinical data warehouses. Data files consisted of 6 tables containing aggregate patient counts for demographic characteristics, clinical course, daily counts, medication class, diagnosis, and laboratory values with mean (SD) (eFigure 1 in Supplement 1). All sites reported diagnosis as *International Statistical Classification of Diseases and Related Health Problems, Tenth Revision* (*ICD-10*) codes and used logical identifier names and codes (LOINC) for laboratory tests and anatomical therapeutic chemical National Drug Codes for medications. Each contributing site uploaded their files to a central 4CE data upload tool, where quality control and validation steps were performed before analysis.^11^ Patient-level files remained at each site and were not centrally shared at any point.

### Demographic and Clinical Variables

Race and ethnicity data were collected by participating hospitals based on routine practices using local race and ethnicity classifications. Sites mapped these categories to the standard categories provided by the US National Institutes of Health before the file upload to 4CE.^12^ We chose to assess race and ethnicity in this study because prior reports have indicated an association between race and ethnicity and clinical outcomes for children and youth with COVID-19.^13,14,15^

A set of 16 laboratory values were selected, reflecting laboratory tests commonly performed as well as tests reported in prior studies to be abnormal in patients with COVID-19.^16^ To describe clinical complications, we analyzed all diagnostic codes assigned to patients during the hospitalization. The diagnosis codes were reported from all sites using *ICD-10*. These codes were truncated to the first 3 characters, which represent the disease category. The codes that follow the first 3 characters add more detailed information about etiology, anatomic site, or manifestations, but would have resulted in too many categories with very low counts. To assess medication use, we determined the number of patients treated with a prespecified set of medications. These included repurposed agents used to manage COVID-19 during the study period (eg, hydroxychloroquine), investigational agents (eg, remdesivir), and adjunctive therapies used to manage complications related to COVID-19.^17^

### Quality-Control Procedures

Validation steps were performed to check that file and column names were correct, columns were in the correct order, values used the correct codes or were within allowed ranges, and that there were no duplicate records. An R script was run to perform additional quality control, including ensuring the 3-digit diagnosis codes were consistent with the *ICD* dictionary. Because all laboratory tests were mapped to the same LOINC codes with unified units, laboratory test values from each site were manually reviewed to ensure the result ranges were generally consistent with data observed across other sites. Sites with implausible laboratory values or values consistently lower or higher than other sites were contacted for further investigation and correction as needed.^11^ The local investigations and final assessment of accepted values considered age-specific reference ranges as well as clinical assays and site ranges.

### Statistical Analysis

We summarized the daily hospitalized case counts over time and the breakdown of the cases by demographic subgroups based on pooled analysis across participating hospitals by country. To describe the clinical profile of hospitalized cases, we reported mean laboratory values at admission and percentages of frequently observed complications. Mean values and percentages with 95% CIs were aggregated across all sites based on random-effects meta-analysis.^18^ To summarize temporal trends of laboratory values, we combined data from sites with at least 3 observations and calculated mean laboratory values on each day of hospitalization, also using random-effects meta-analysis.^18^ Additional details on this approach are provided in the eMethods and eFigures 2 and 3 in Supplement 1. We based 95% CIs on the *z*-statistic with normal approximations for both continuous outcomes and the proportion of binary outcomes. Statistical significance was prespecified at *P* < .05 and tests were 2-tailed.

Statistical analyses and visualizations were performed in R version 3.5.1 (R Project for Statistical Computing) and Python version 3.7 (Python). We used the Altair package^19^ to create figures for static publication and interactive web-based exploration. The Structured Query Language code used for data extraction, R Code used for analysis, and mapping tables used for laboratory tests and medications are available on GitHub.

## Results

### Study Cohort

**Figure 1.**
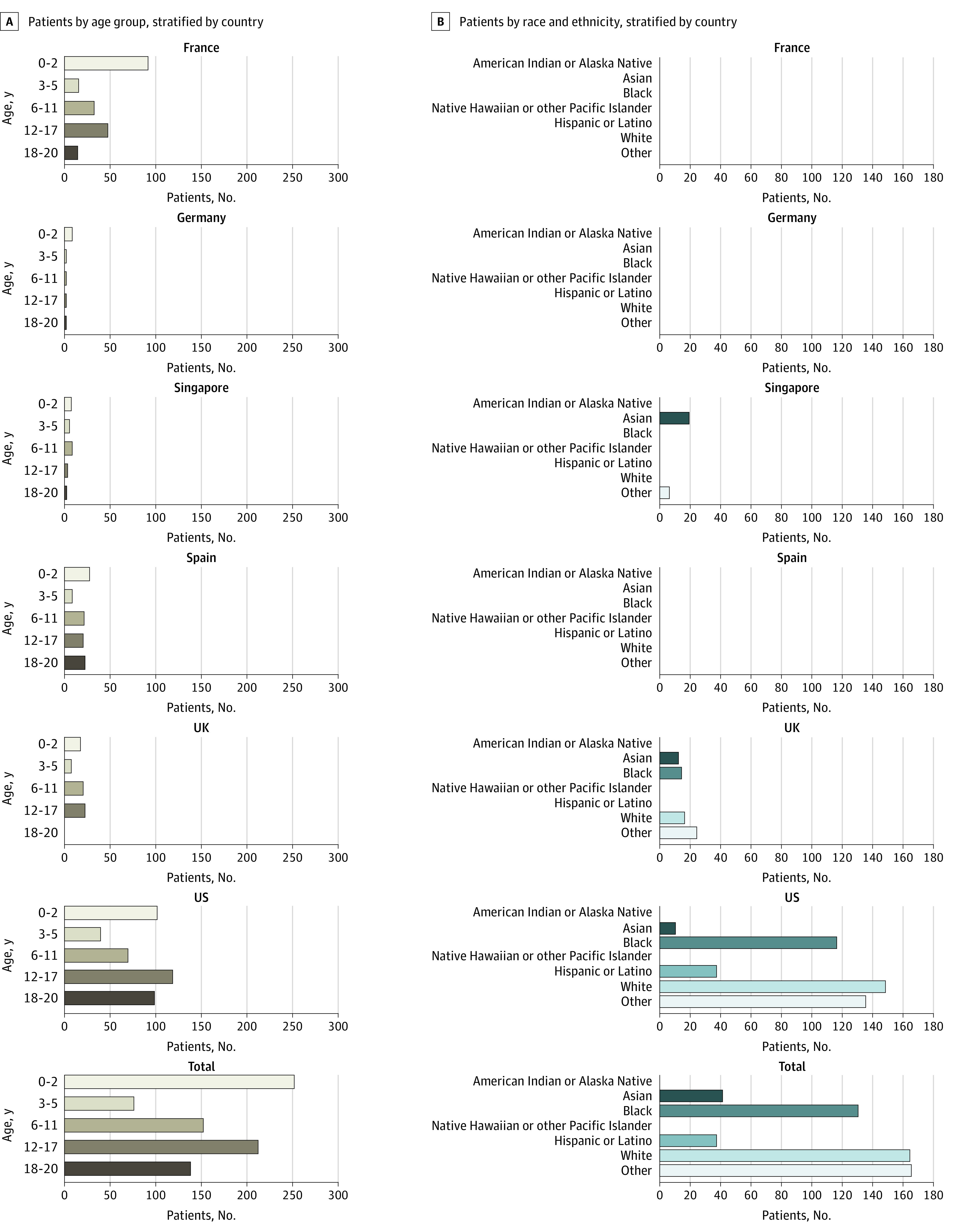
Age and Race/Ethnicity Distribution by Country The race/ethnicity variable was based on the categories as defined by the US National Institutes of Health.^12^ For Singapore, the term Asian includes Chinese, Asian Indian, and Malaysian and the term other was used for Eurasian and other races and ethnicities. For patients in the UK and the US, the term other represents other races and ethnicities, mixed races, and missing information on race. Information on race and ethnicity were not collected in France, Germany, and Spain.

There were 347 male patients (52%; 95% CI, 48.5%-55.3%) and 324 female patients (48%; 95% CI, 44.4%-51.3%) in our cohort. There was a bimodal age distribution, with the greatest proportion of patients in the 0- to 2-year (199 patients [30%]) and 12- to 17-year (170 patients [25%]) age range (Figure 1). Race and ethnicity data were not collected by sites in France, Germany, and Spain in accordance with national practices and standards. For Singapore, only 1 option was provided for Asian race, thus all local Asian groups were classified as Asian patients.

Data were collected on 671 hospitalized children and youth with PCR-confirmed SARS-CoV-2 infection across a total of 27 hospitals in 6 countries, including in France (4 hospitals), Germany (1 hospital), Singapore (1 hospital), Spain (1 hospital), the UK (1 hospital), and the US (19 hospitals). Pediatric cases were identified at each site, with France contributing 145 cases, Germany, 8 cases; Singapore, 24 cases; Spain, 78 cases; the UK, 62 cases; and the US, 354 cases.

**Figure 2.**
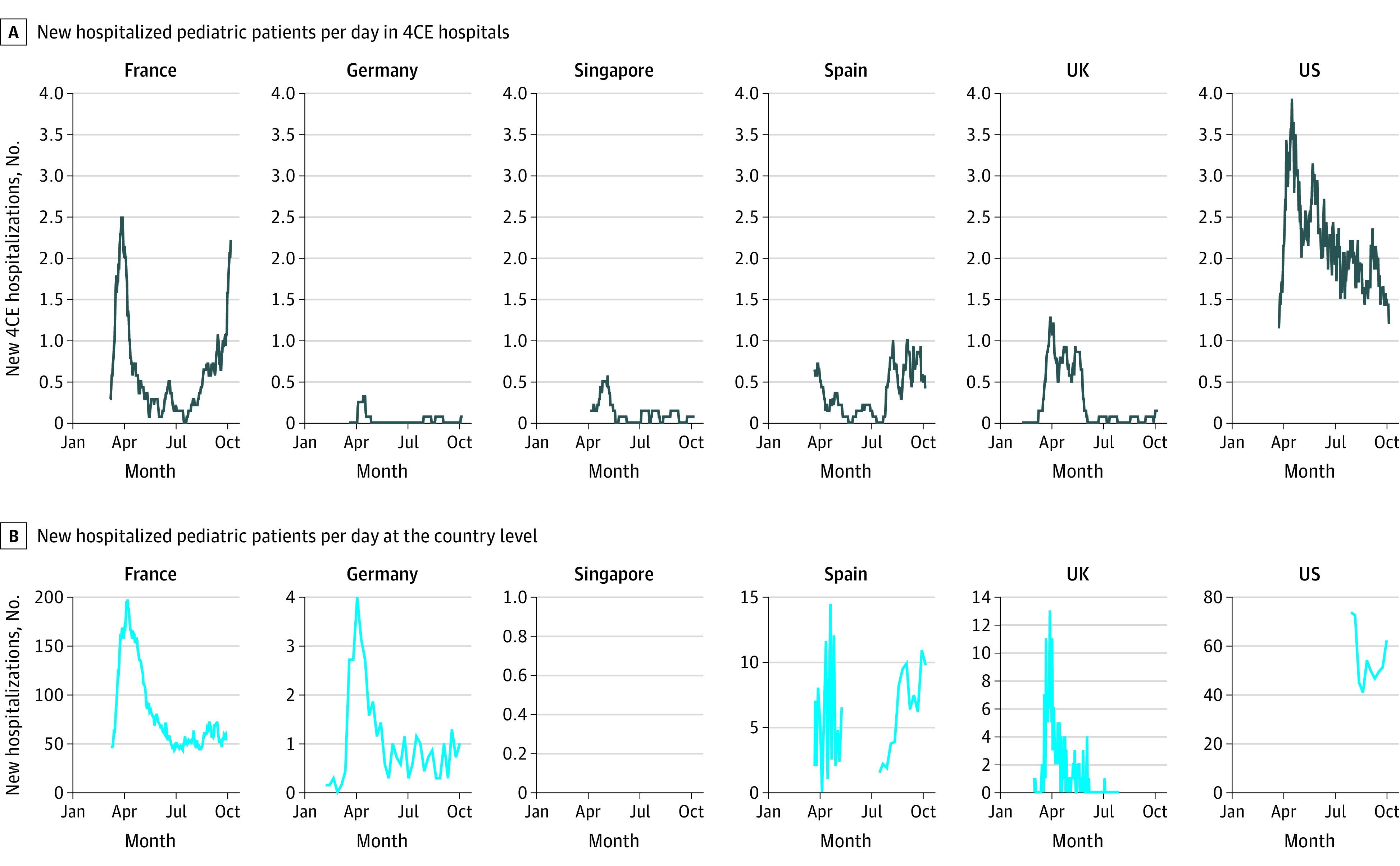
Hospitalized Pediatric Case Counts by Country For France, daily pediatric hospitalization data were obtained from Santé Publique France.^20^ For Germany, weekly pediatric hospitalization data were obtained from the German Society for Pediatric Infectious Diseases.^21^ National pediatric hospitalization data were not available for Singapore. For Spain, weekly pediatric hospitalization data were obtained from the Spanish National Epidemiological Surveillance Network, which lacks hospitalization counts between May 11 and July 15, 2020.^22^ For the UK, daily pediatric hospitalization data were obtained from the Royal College of Paediatrics and Child Health and represent pediatric hospitalizations in England.^23^ For the US, weekly pediatric hospitalization data between July 31, 2020, and October 9, 2020, were obtained from the Department of Health and Human Services.^24^ The y-axis scales for country-level data are independent to compare country-level trends with Consortium for Clinical Characterization of COVID-19 by EHR (4CE) trends. The plots in Figure 2A display the counts with a 14-day (centered) rolling mean.

Figure 2 illustrates the number of hospitalized pediatric patients by date during the study period.^20,21,22,23,24^ Trends demonstrated discrete surges in hospitalization counts, with most countries experiencing a distinct increase in pediatric hospitalizations in the spring followed by variations in the occurrence and timing of subsequent surges. National-level data on pediatric hospitalizations for France, Germany, Spain, and the UK mirrored the 4CE hospitalization data. For example, peaks during the spring of 2020 in national-level data and 4CE hospitalizations occurred 19 days apart in France and 11 days apart in Germany. The largest difference was in Spain, with peaks during the initial spring surge occurring 23 days apart. National-level data were not available for Singapore and only available for a 2-month period for the US. Additional visualizations, including interactive figures with cumulative counts by country are available online.^25^

### Clinical Features

A total of 27 364 laboratory values were obtained for the 16 laboratory tests examined (Table 1). Mean values across hospitals were abnormal at the time of admission for markers of inflammation and coagulation. Specifically, C-reactive protein was elevated to 83 mg/L (95% CI, 53-112 mg/L; to convert to milligrams per liter, multiply by 10), ferritin to 417 ng/mL (95% CI, 228-607 ng/mL; to convert to micrograms per liter, multiply by 1), and procalcitonin to 1.45 ng/mL (95% CI, 0.13-2.77 ng/mL). However, mean values for both white blood cell count and neutrophil count were within normal limits. Dimerized plasmin fragment D (D-dimer) was elevated to 0.78 μg/mL (95% CI, 0.35-1.21 μg/mL; to convert to nanomoles per liter, multiply by 5.476) and fibrinogen to 477 mg/dL (95% CI, 385-569 mg/dL; to convert to grams per liter, multiply by .01). In a subset of patients (n = 59), cardiac troponin was elevated to 0.032 ng/mL (95% CI, 0.000-0.080 ng/mL; to convert to milligrams per liter, multiply by 1.0). Common complications included cardiac arrhythmias (15.0%; 95% CI, 8.1%-21.7%), viral pneumonia (13.3%; 95% CI, 6.5%-20.1%), and respiratory failure (10.5%; 95% CI, 5.8%-15.3%). The total number of deaths across participating sites was 18 (2.7%).

**Table 1.** Clinical Features of Children and Youth Hospitalized With COVID-19

Characteristic	Values at admission, mean (95% CI)
Laboratory results^**a**^	
Alanine aminotransferase (reference range, 0-41 U/L)	33.2 (28.8-37.6) U/L
Albumin (reference range, 2.5-5 g/dL)	3.7 (3.4-4.0) g/dL
Aspartate aminotransferase (reference range, 8-92 U/L)	44 (34-55) U/L
Cardiac troponin (reference range, 0-0.01 ng/mL)	0.032 (0.000-0.080) ng/mL
C-reactive protein (reference range, 0-5 mg/L)	83 (53-112) mg/L
Creatinine (reference range, 0.1-1.0 mg/dL)	0.65 (0.59-0.70) mg/dL
D-dimer (reference range, 0-500 μg/mL)	0.78 (0.35-1.21) μg/mL
Ferritin (reference range, 12-200 ng/mL)	417 (228-607) ng/mL
Fibrinogen (reference range, 150-400 mg/dL)	477 (385-569) mg/dL
Lactate dehydrogenase (reference range, 400-1300 U/L)	449 (385-569) U/L
Lymphocyte count (reference range, 1.5-10 K/μL)	1.9 (1.7-2.2) K/μL
Neutrophil count (reference range, 1.5-10 K/μL)	6.2 (5.6-6.7) K/μL
Procalcitonin (reference range, 0-0.15 ng/mL)	1.45 (0.13-2.77) ng/mL
Prothrombin time (reference range, 8.5-17 s)	14.4 (13.3-15.4) s
Total bilirubin (reference range, 0-1.2 mg/dL)	0.76 (0.55-0.97) mg/dL
White blood cell count (reference range, 5-26 K/μL)	9.4 (8.7-10.0) K/μL
Complications, proportion of patients during hospitalization (95% CI)	
Cardiac arrhythmias	15.0 (8.1-21.7)
Viral Pneumonia	13.3 (6.5-20.1)
Respiratory failure	10.5 (5.8-15.3)
Shock	9.5 (3.7-15.3)
Acute kidney failure	7.2 (3.5-10.8)
Pleural effusion	6.6 (3.0-10.2)
Length of hospitalization, mean (95% CI), d	11.1 (8.1-14.2)

Abbreviation: D-dimer, dimerized plasmin fragment D.

SI conversion factors: To convert alanine aminotransferase to microkatal per liter, multiply by 0.0167; albumin to g/L, multiply by 10; aspartate aminotransferase to microkatal per liter, multiply by 0.0167; C-reactive protein to milligrams per liter; creatinine to micromoles per liter, multiply by 76.25; ferritin to micrograms per liter, multiply by 1; D-dimer to nanomoles per liter, multiply by 5.476; fibrinogen to grams per liter, multiply by .01; lactate dehydrogenase to microkatal per liter, multiply by 0.0167; lymphocyte count to proportion of 1.0, multiply by 0.01; neutrophil to proportion of 1.0, multiply by 0.01; total bilirubin to micromoles per liter, multiply by 17.104; troponin to milligrams per liter, multiply by 1.0, white blood cell count to proportion of 1.0, multiply by 0.01.

^a^ Reference ranges as reported in The Royal College of Paediatrics and Child Health.

**Figure 3.**
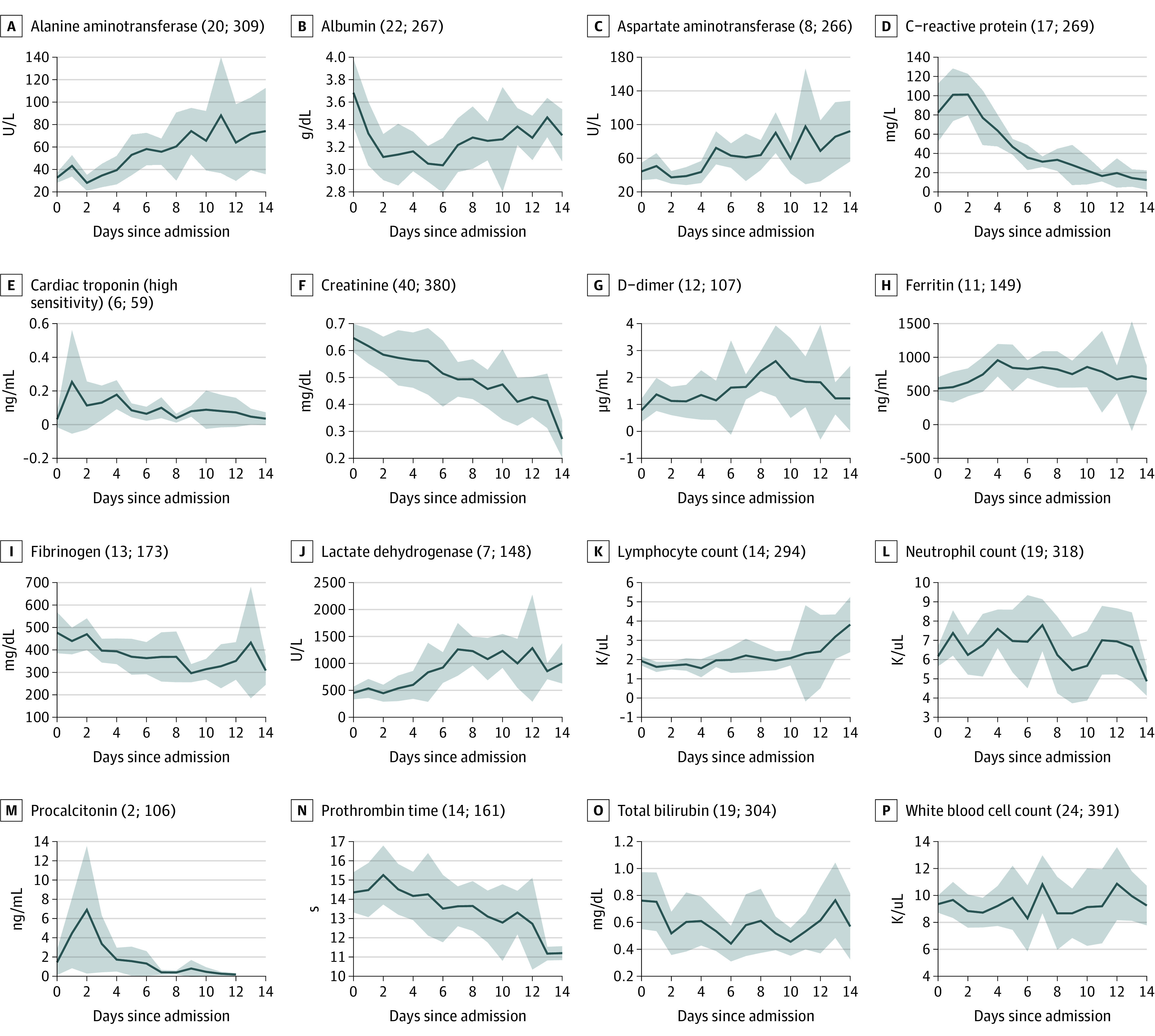
Trajectories for Laboratory Values During Hospitalization Mean daily values across sites were calculated using random-effects meta-analysis. Values in parenthesis represent the minimum and maximum numbers of patients contributing data on any single day during the 14-day observation period. The shaded areas represent 95% CIs. SI conversion factors: To convert alanine aminotransferase to microkatal per liter, multiply by 0.0167; albumin to g/L, multiply by 10; aspartate aminotransferase to microkatal per liter, multiply by 0.0167; C-reactive protein to milligrams per liter; creatinine to micromoles per liter, multiply by 76.25; ferritin to micrograms per liter, multiply by 1; D-dimer to nanomoles per liter, multiply by 5.476; fibrinogen to grams per liter, multiply by .01; lactate dehydrogenase to microkatal per liter, multiply by 0.0167; lymphocyte count to proportion of 1.0, multiply by 0.01; neutrophil to proportion of 1.0, multiply by 0.01; total bilirubin to micromoles per liter, multiply by 17.104; troponin to milligrams per liter, multiply by 1.0, white blood cell count to proportion of 1.0, multiply by 0.01.

Figure 3 illustrates trajectories of laboratory values for the first 14 sequential days since admission. The number of patients with laboratory tests was highest during the initial days of hospitalization with subsequent decreases for all laboratory tests over the course of hospitalization (eFigure 3 in Supplement 1). Although the selective ordering of laboratory tests based on patients’ condition limits interpretation, markers of inflammation (C-reactive protein, ferritin, neutrophil count, procalcitonin) that were initially elevated generally showed improvement after hospital days 2 to 4. For example, compared with the initial values for C-reactive protein, 4-day measurements showed a decrease of 18 mg/L (95% CI, −16-54 mg/L). Interestingly, there was a peak in several laboratory values, such as albumin, D-dimer, and lactate dehydrogenase, starting on hospital days 6 to 8. For example, compared with the initial values for D-dimer, 8-day measurements showed an increase of 1.45 μg/mL (95% CI, 0.59-2.31 μg/mL).

### Medication Use

To examine the use of specific drug classes in the treatment of COVID-19 in children and youth, we determined the number of sites treating at least 3 patients with a range of drugs considered candidate therapeutic agents in adults during the study period or used to manage certain complications and underlying conditions potentially exacerbated by COVID-19 (Table 2). Only 2 sites treated at least 3 patients with an aminoquinoline, which includes hydroxychloroquine, and 1 site administered remdesivir to at least 3 patients. More sites administered adjunctive therapies, such as antithrombotic agents (8 sites), diuretics (8 sites), interleukin inhibitors (3 sites), and angiotensin converting enzyme inhibitors (3 sites).

**Table 2.** Medication Use

Drug class^a^	Country using the drugs	Health care sites treating at least 3 patients, No, (%) (n = 14)^b^	Patients treated, No.
Repurposed agents			
Aminoquinolines	US	2 (14)	7
Antivirals for HIV and other viral infections	None	0	0
Interferons	None	0	0
Investigational agents			
Remdesivir	UK	1 (7)	6
Adjunctive therapies			
Antithrombotic agents	France, UK, US	8 (57)	137
Diuretics	France, UK, US	8 (57)	97
Interleukin inhibitors	UK, US	3 (21)	12
Angiotensin-converting enzyme inhibitors	US	3 (21)	12
Angiotensin II receptor blockers	None	0	0

^a^ Antivirals include lopinavir and ritonavir, darunavir, and ribavirin; aminoquinolines include hydroxychloroquine and chloroquine; interferons include interferon β-1b and peginterferon alfa-2a; and interleukin inhibitors include anakinra, sarilumab, tocilizumab, and siltuximab.

^b^ The 27 participating hospitals represent 14 health care sites contributing aggregate data. Hospital-level data are reported according to health care sites, precluding analysis of the number of hospitals providing specific therapies.

## Discussion

Using patient-level EHR data extracted from health care systems across 6 countries, this study offers insights on international trends of hospitalizations for children and youth with COVID-19 and defines epidemiological and clinical features associated with the disease in children and youth. Even among countries with few participating sites, hospitalization counts for children and youth over an 8 month period approximated population-level infection rates, demonstrating the potential application of this approach to monitoring disease activity in pediatric populations. Consistent with prior reports, we found greater proportions of younger children among hospitalized patients.^26,27^ Laboratory tests obtained on hospital admission indicated abnormalities in inflammation and coagulation. Examination of management patterns revealed that the use of candidate therapeutic agents adopted in adult populations remained low in children and youth.

Our study demonstrates the value of using routinely collected data from EHRs to complement other forms of disease surveillance, especially when disease prevalence is low and rapid progression precludes the development of prospective research infrastructures.^28^ These data may be particularly valuable in advancing our understanding of COVID-19 in children and youth, where fewer resources have focused on COVID-19–related illness because of the less severe impact of the disease and much lower disease prevalence. While there are important limitations to EHR data, including inconsistent and incomplete recording of certain data elements, 4CE demonstrates how contemporary informatics methods can enable efficient integration and analysis of large volumes of clinical information to build observational data sets across health care systems and countries. A unique feature of this EHR-based network is the rapid onboarding facilitated by the open-source i2b2 and OMOP software platforms, which allowed a total of 96 adult and pediatric hospitals to join during an initial 2-week period. Rapid availability of this type of curated EHR data can support hypothesis generation and prioritization of clinical trials, understanding of the natural course of disease, identification of rare complications and phenotypes, and anticipatory planning by health care institutions around resource requirements and medical supply needs.

Laboratory values were extracted for a core set of tests to support a detailed assessment of the clinical course of patients with COVID-19. Laboratory results are typically not available in administrative or medical claims data sets, which are limited to information on test ordering. We collected daily laboratory values for each day of hospitalization to build trajectories for individual tests. An extensive quality control process was performed to address any mapping errors between sites. However, values should be interpreted with some caution because tests are not obtained consistently on all patients and reflect physician decisions based on the patient’s clinical presentation and local health care workflows.^29^ Evaluation of the number of patients with available laboratory tests indicated that the number tested dropped quickly during the first week of hospitalization. Nonetheless, our findings were consistent with clinical results presented in case series and meta-analyses, showing generally normal white blood cell counts and abnormally elevated inflammatory markers and coagulation tests.^30,31,32,33^ Laboratory trajectories also revealed a gradual decline in the value of certain inflammatory markers during hospitalization. Interestingly, several laboratory tests demonstrated increased values beginning around the second week of hospitalization, such as kidney and liver function markers. Additional studies will be needed to determine whether these laboratory tests can predict specific disease trajectories and complications among hospitalized children and youth.

The multinational design of 4CE allows ascertainment of differences in regional management patterns and uptake of therapeutic interventions in children and youth. Early in the pandemic, many agents emerged as candidate therapies for COVID-19, including both repurposed drugs and investigational agents.^17^ Observational studies^31,34,35,36,37,38^ indicate that many of these drugs were widely used among hospitalized adult patients, although use in children and youth appears to have been lower. This likely reflects the less severe disease course in children and youth and is also consistent with patterns in off-label medication prescribing in pediatric patients, where use in pediatric populations tends to follow adoption in adults.^39^ It also relates to the lower number of clinical trials performed in pediatric patients to test new therapies, including remdesivir.^2^ Monitoring the use of pharmacotherapies in children and youth, including defining regional and country-level differences, will support activities to optimize and standardize care for children and youth with COVID-19 and guide prioritization of research activities to ensure availability of safe and effective pediatric therapies.

An area for further development of 4CE data is in the collection and analysis of race and ethnicity information. During this first phase of data collection, the race variable was limited to standard categories as defined by the US National Institutes of Health and how it is used in many US-based studies.^12^ However, this categorization is subject to 2 major limitations for our purposes. First, it combines race and ethnicity in a way that race cannot be reported for Hispanic and Latino individuals. This results in missing race information if a patient is recorded as Hispanic or Latino or missing ethnicity data if race is prioritized in local data collection. Second, this categorization does not lend itself to use in other countries, such as the UK or Singapore, where the primary racial and ethnic categories differ from those in the US. To meaningfully capture race and ethnicity information across countries, country-specific categories must be used. Accurate collection of race and ethnicity information is critical to advancing our understanding of differences in risk factors, infection rates, and health care use that have been reported in prior studies.^15,40,41^ In future phases of 4CE, we plan to implement country-specific ontologies for collection of race and ethnicity data.

### Limitations

This study has limitations. To enable an international federated network and preserve patient data privacy from each participating site, only aggregate counts were analyzed, limiting the ability to combine values or follow individual patients longitudinally. For example, while we can ascertain mean laboratory values across individual sites and even track these throughout the hospitalization, we cannot link laboratory results to specific patient characteristics. In the next phase of 4CE studies, prespecified analyses will be run within the primary data sets at each of the individual sites before aggregation at the consortium-level, enabling patient-level analyses. Additional limitations relate to the use of observational data, including nonsystematic recording of certain clinical data elements and shifting testing strategies for COVID-19, which may inform characteristics of the study population.^29^

## Conclusions

In this study of EHRs of children and youth hospitalized with COVID-19 in 6 countries, we demonstrated country-level variation in trends in COVID-19 hospitalization for children and youth and defined clinical complications and laboratory test abnormalities. Large-scale informatics-based approaches can be applied to complement other methods of disease surveillance and define epidemiological and clinical features of COVID-19 in children and youth. Further study and use of EHR informatics-based efforts may facilitate improved modeling of pediatric COVID-19 trajectories and inform clinical care for pediatric patients.

## Notes

Supplement 1. **eTable.** Contributing sites **eFigure 1.** Federated data collection across participating sites **eFigure 2.** Forest plots for laboratory values at admission **eFigure 3.** Sample size plot **eMethods.** Calculation of temporal trends for laboratory values

Supplement 2. **Nonauthor Collaborators.** The Consortium for Clinical Characterization of COVID-19 by EHR (4CE) coordinators and investigators
