# Errors in Byline

**DOI:** 10.1001/jamanetworkopen.2021.22388

**Published:** 2021-07-23

In the Original Investigation titled “International Analysis of Electronic Health Records of Children and Youth Hospitalized with COVID-19 Infection in 6 Countries,”^1^ published June 11, 2021, there were hyphens missing in the surnames of 4 authors in the byline. The names have been updated to Jaime Cruz-Rojo, MD; Noelia García-Barrio, MS; Miguel Pedrera-Jiménez, MS; and Pablo Serrano-Balazote, MD, MS. Additionally, the degree was corrected for Tianxi Cai, ScD, in the byline. This article has been corrected.^1^
