# Supplementary material for: International Analysis of Electronic Health Records of Children and Youth Hospitalized With COVID-19 Infection in 6 Countries
Source: JAMA Netw Open. 2021 Jun 11;4(6):e2112596. doi: 10.1001/jamanetworkopen.2021.12596 (PMC8196345; doi:10.1001/jamanetworkopen.2021.12596)
Supplement: Supplement 1. — eTable. Contributing sites eFigure 1. Federated data collection across participating sites eFigure 2. Forest plots for laboratory values at admission eFigure 3. Sample size plot eMethods. Calculation of temporal trends for laboratory values [file jamanetwopen-e2112596-s001.pdf]

Supplemental Online Content

Bourgeois FT, Gutiérrez-Sacristán A, Keller MS, et al; Consortium for Clinical Characterization of COVID-19 by EHR (4CE). International analysis of electronic health records of children and youth hospitalized with COVID-19 infection in 6 countries. *JAMA Netw Open*. 2021;4(6):e2112596. doi:10.1001/jamanetworkopen.2021.12596

eTable. Contributing sites
eFigure 1. Federated data collection across participating sites
eFigure 2. Forest plots for laboratory values at admission
eFigure 3. Sample size plot
eMethods. Calculation of temporal trends for laboratory values

This supplemental material has been provided by the authors to give readers additional information about their work.

© 2021 Bourgeois FT et al. *JAMA Network Open*.

eTable1. Contributing Sites

Country	Sites: Healthcare System	City	Hospitals per site	Pediatric beds	Pediatrics discharges per year	Obfuscation threshold	Obfuscation blurring range
France	Assistance Publique - Hôpitaux de Paris	Paris	3	1,207	58,988	<3	none
	Bordeaux University Hospital	Bordeaux	1	251	20,889	none	none
USA	University of Kansas Medical Center	Kansas City, KS	1	19	6,500	<4	none
	Mass General Brigham (Partners Healthcare)	Boston, MA	8	150	12,000	<3	+/-3
	University of Michigan	Ann Arbor, MI	1	239	8,252	none	none
	University of North Carolina at Chapel Hill	Chapel Hill, NC	1	300	19,172	<10	none
	Northwestern University	Chicago, IL	5	193	15,748	none	none
	Boston Children's Hospital	Boston, MA	1	404	28,000	none	none
	The Children's Hospital of Philadelphia	Philadelphia, PA	1	564	25,406	none	none
	University of Pittsburgh / UPMC	Pittsburgh, PA	1	313	12,707	none	none
Singapore	National University Hospital	Singapore	1	730	17,511	none	none
Germany	Medical Center, University of Freiburg	Freiburg	1	124	9,000	<4	none
UK	Great Ormond Street Hospital for Children	London	1	423	40,273	<3	none
Spain	Hospital Universitario 12 de Octubre	Madrid	1	83	5,572	<3	none

© 2021 Bourgeois FT et al. *JAMA Network Open*.

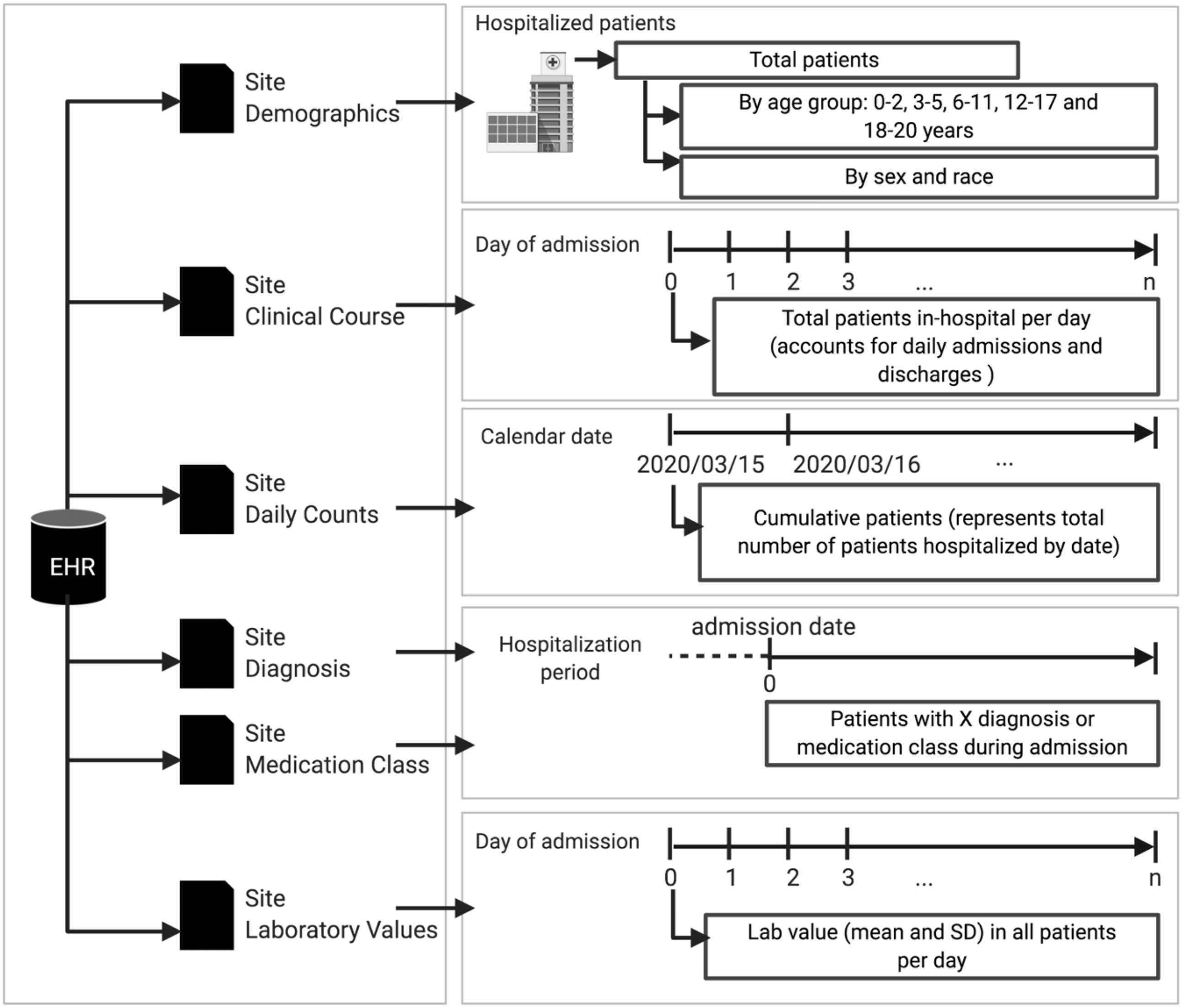

eFigure 1. Federated data collection across participating sites. Each site generated six data tables containing aggregate counts and statistics on their patient-level data: 1) demographic breakdowns, 2) clinical course accounts for daily admissions and discharges, 3) cumulative counts of total hospitalized patients, 4) diagnosis counts during hospitalization, 5) medication class counts during hospitalization, and 6) daily trajectories of lab tests. These aggregate descriptive files were provided to the 4CE consortium. This figure has been created with BioRender.com

© 2021 Bourgeois FT et al. *JAMA Network Open*.

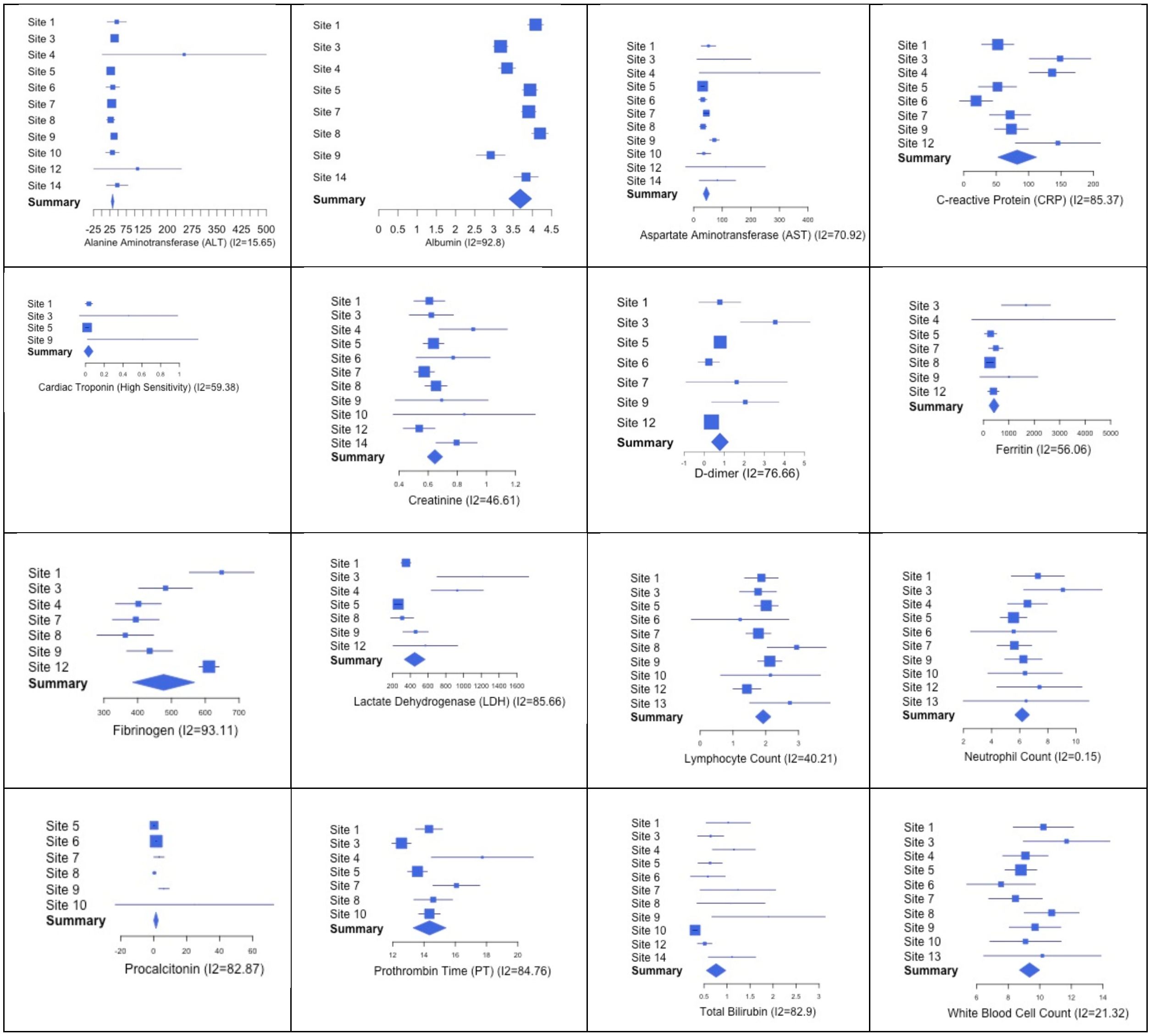

eFigure 2. Forest plots for laboratory values at admission. Plots indicate mean values at admission with 95% confidence intervals based on Z-statistic at each site. The heterogeneous structure across different sites supported a random-effects meta-analysis for the statistical model. The aggregated mean values (Summary) and 95% confidence intervals in each forest plot were based on random-effects meta-analysis.

© 2021 Bourgeois FT et al. *JAMA Network Open*.

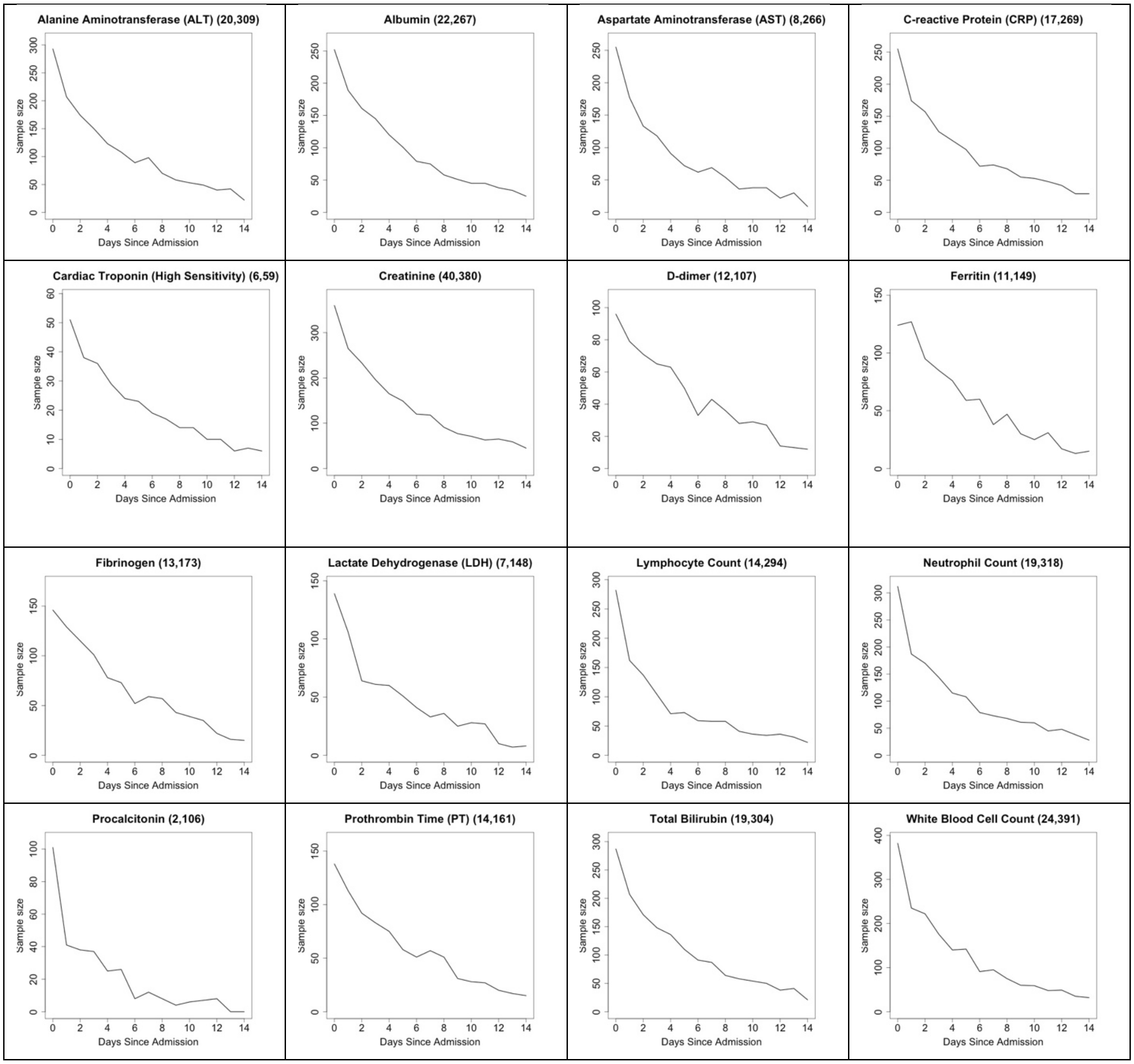

eFigure 3. Plots of daily sample sizes for the temporal trend analyses over first 14 days of hospitalizations.

© 2021 Bourgeois FT et al. *JAMA Network Open*.

eMethods. Calculation of temporal trends for laboratory values

To summarize the temporal trends of laboratory tests, we combined data from sites with at least three observations and analyzed temporal patterns by calculating mean laboratory values on each day of hospitalization, also using random-effects meta-analysis. Specifically, for a specific laboratory test and on each day of hospitalization, we conducted a random-effect meta-analysis using the site level mean and standard error (SE) as input to obtain an overall mean and SE estimate. We then ploted these estimates over the first 14 days of hospitalization.

© 2021 Bourgeois FT et al. *JAMA Network Open*.

© 2021 Bourgeois FT et al. *JAMA Network Open*.
