# Supplementary material for: International Analysis of Electronic Health Records of Children and Youth Hospitalized With COVID-19 Infection in 6 Countries
Source: JAMA Netw Open. 2021 Jun 11;4(6):e2112596. doi: 10.1001/jamanetworkopen.2021.12596 (PMC8196345; doi:10.1001/jamanetworkopen.2021.12596)
Supplement: Supplement 2. — Nonauthor Collaborators. The Consortium for Clinical Characterization of COVID-19 by EHR (4CE) coordinators and investigators [file jamanetwopen-e2112596-s002.pdf]

Supplemental Online Content: Nonauthor Collaborators

*Indicates required information. Only first name, last name, and suffix will appear in PubMed.

| *Group Name(s): Consortium for Clinical Characterization of COVID-19 by EHR (4CE) | | | | | | | |
|---|---|---|---|---|---|---|---|
| ***First Name and Middle Initial(s)** | ***Last Name** | ***Suffix (eg, Jr, III)** | Academic Degrees | Institution | Location (city, state/province, country) | Role or Contribution, eg, chair, principal investigator | Group (if more than 1 Group listed in the byline) and/or Subgroup (eg, Steering Committee) |
| James R | Aaron | | MHA | University of Kentucky | Lexington, KY, United States of America | Member | |
| Giuseppe | Agapito | | PhD | University Magna Graecia of Catanzaro, Italy | Catanzaro, ITALY | Member | |
| Adem | Albayrak | | | Health Catalyst, INC. | Cambridge, MA, United States of America | Member | |
| Mario | Alessiani | | MD, FACS | ASST Pavia, Lombardia Region Health System | Pavia, Italy | Member | |
| Danilo F | Amendola | | MD | Clinical Research Unit of Botucatu Medical School, São Paulo State University, Botucatu, Brazil | Botucatu, Brazil | Member | |
| François | Angoulvant | | MD,PhD | Hôpital Necker-Enfants Malades, Assistance Public-Hôpitaux de Paris, Paris | Paris, France | Member | |
| Li LLJ | Anthony | | | National Center for Infectious Diseases, Tan Tock Seng Hospital, Singapore | Novena, Singapore | Member | |
| Andrew | Atz | | MD | Medical University of South Carolina | Charleston, SC, United States of America | Member | |
| James | Balshi | | | St. Luke's University Health Network, Bethlehm PA | Bethlehem, PA, United States of America | Member | |
| Brett K | Beaulieu-Jones | | PhD | Harvard Medical School | Boston, MA, United States of America | Member | |
| Douglas S | Bell | | | David Geffen School of Medicine at UCLA | Los Angeles CA, United States of America | Member | |
| Antonio | Bellasi | | | UOC Ricerca, Innovazione e Brand reputation, ASST Papa Giovanni XXIII, Bergamo | Bergamo, Italy | Member | |
| Riccardo | Bellazzi | | MS, PhD | University of Pavia, Italy | Pavia, Italy | Member | |
| Vincent | Benoit | | PhD | APHP Greater Paris University Hospital | Paris, France | Member | |
| Michele | Beraghi | | | ASST Pavia | Voghera (PV), Italy | Member | |
| José Luis | Bernal Sobrino | | MS | Hospital Universitario 12 de Octubre, Madrid, Spain | Madrid, Spain | Member | |
| Mélodie | Bernaux | | | APHP Greater Paris University Hospital | Paris, France | Member | |
| Romain | Bey | | | APHP Greater Paris University Hospital | Paris, France | Member | |

Supplemental Online Content: Nonauthor Collaborators

*Indicates required information. Only first name, last name, and suffix will appear in PubMed.

| *First Name and Middle Initial(s) | *Last Name | *Suffix (eg, Jr, III) | Academic Degrees | Institution | Location (city, state/province, country) | Role or Contribution, eg, chair, principal investigator | Group (if more than 1 Group listed in the byline) and/or Subgroup (eg, Steering Committee) |
|---|---|---|---|---|---|---|---|
| Alvar | Blanco Martínez | | MS | Hospital Universitario 12 de Octubre, Madrid, Spain | Madrid, Spain | Member | |
| Silvano | Bosari | | Prof. | IRCCS Ca' Granda Ospedale Maggiore Policlinico di Milano | Milan, Italy | Member | |
| Robert L | Bradford | | | North Carolina Translational and Clinical Sciences (NC TraCS) Institute, UNC Chapel Hill | Chapel Hill, NC, United States of America | Member | |
| Gabriel A | Brat | | MD | Harvard Medical School | Boston, MA, United States of America | Member | |
| Stéphane | Bréant | | | APHP Greater Paris University Hospital | Paris, France | Member | |
| Nicholas W | Brown | | MEng | Harvard Medical School | Boston, MA, United States of America | Member | |
| William A | Bryant | | PhD | Great Ormond Street Hospital for Children, UK | London WC1N 3JH, UK | Member | |
| Mauro | Bucalo | | MS | BIOMERIS (BIOMedical Research Informatics Solutions) | Pavia, Italy | Member | |
| Anita | Burgun | | | APHP Greater Paris University Hospital | Paris, France | Member | |
| Mario | Cannataro | | M.Sc. | University Magna Graecia of Catanzaro, Italy | Catanzaro, ITALY | Member | |
| Aldo | Carmona | | | St. Luke's University Health Network, Bethlehem, PA | Bethlehem, PA, United States of America | Member | |
| Charlotte | Caucheteux | | | Université Paris-Saclay, Inria, CEA | Palaiseau, France | Member | |
| Julien | Champ | | | INRIA Sophia-Antipolis – ZENITH team, LIRMM, Montpellier, France | Montpellier Cedex 5, France | Member | |
| Krista | Chen | | BS | Boston Children's Hospital | Boston, MA, United States of America | Member | |
| Jin | Chen | | PhD | University of Kentucky | Lexington, KY, United States of America | Member | |
| Luca | Chiovato | | | Unit of Internal Medicine and Endocrinology, Istituti Clinici Scientifici Maugeri SpA SB IRCCS, Pavia, Italy and Department of Internal Medicine and Therapeutics, University of Pavia, Pavia, Italy. | Pavia, Italy | Member | |
| Lorenzo | Chiudinelli | | PhD | ASST Papa Giovanni XXIII, Bergamo | Bergamo, Italy | Member | |
| James J | Cimino | | MD | University of Alabama at Birmingham | Birmingham, Alabama, United States of America | Member | |
| Tiago K | Colicchio | | PhD, MBA | University of Alabama at Birmingham | Birmingham, Alabama, United States of America | Member | |

*Indicates required information. Only first name, last name, and suffix will appear in PubMed.

| ***First Name and Middle Initial(s)** | ***Last Name** | ***Suffix (eg, Jr, III)** | Academic Degrees | Institution | Location (city, state/province, country) | Role or Contribution, eg, chair, principal investigator | Group (if more than 1 Group listed in the byline) and/or Subgroup (eg, Steering Committee) |
|---|---|---|---|---|---|---|---|
| Sylvie | Cormont | | | APHP Greater Paris University Hospital | Paris, France | Member | |
| Sébastien | Cossin | | | Bordeaux University Hospital / ERIAS - Inserm U1219 BPH | Bordeaux, France | Member | |
| Jean B | Craig | | PhD | Medical University of South Carolina | Charleston, South Carolina, United States of America | Member | |
| Juan Luis | Cruz Bermúdez | | PhD | Hospital Universitario 12 de Octubre, Madrid, Spain | Madrid, Spain | Member | |
| Arianna | Dagliati | | MS, PhD | University of Pavia, Italy | Pavia, Italy | Member | |
| Mohamad | Daniar | | MSIS | Boston Children's Hospital | Boston, MA, United States of America | Member | |
| Christel | Daniel | | | APHP Greater Paris University Hospital, INSERM | Paris, France | Member | |
| Anahita | Davoudi | | PhD | University of Pennsylvania Perelman School of Medicine | Philadelphia, PA, United States of America | Member | |
| Julien | Dubiel | | | APHP Greater Paris University Hospital | Paris, France | Member | |
| Scott L | DuVall | | PhD | VA Salt Lake City Health Care System | Salt Lake CIty, UT, United States of America | Member | |
| Loic | Esteve | | | SED/SIERRA, Inria Centre de Paris | Paris, France | Member | |
| Shirley | Fan | | | University of Michigan | Ann Arbor, MI, United States of America | Member | |
| Robert W | Follett | | | David Geffen School of Medicine at UCLA | Los Angeles, CA, United States of America | Member | |
| Paula SA | Gaiolla | | | Botucatu Medical School, São Paulo State University, Botucatu, Brazil | Botucatu, Brazil | Member | |
| Thomas | Ganslandt | | MD | University Medicine Mannheim, Heidelberg University | Mannheim, Germany | Member | |
| Lana X | Garmire | | PhD | University of Michigan | Ann Arbor, MI, United States of America | Member | |
| Tobias | Gradinger | | MD, BSc | University Medicine Mannheim, Heidelberg University | Mannheim, Germany | Member | |
| Alexandre | Gramfort | | | Université Paris-Saclay, Inria, CEA | Palaiseau, France | Member | |
| Romain | Griffier | | | Bordeaux University Hospital | Bordeaux, France | Member | |
| Nicolas | Griffon | | | APHP Greater Paris University Hospital, INSERM | Paris, France | Member | |
| Olivier | Grisel | | | Université Paris-Saclay, Inria, CEA | Palaiseau, France | Member | |

Supplemental Online Content: Nonauthor Collaborators

*Indicates required information. Only first name, last name, and suffix will appear in PubMed.

| ***First Name and Middle Initial(s)** | ***Last Name** | ***Suffix (eg, Jr, III)** | Academic Degrees | Institution | Location (city, state/province, country) | Role or Contribution, eg, chair, principal investigator | Group (if more than 1 Group listed in the byline) and/or Subgroup (eg, Steering Committee) |
|---|---|---|---|---|---|---|---|
| Christian | Haverkamp | | | Faculty of Medicine and Medical Center, University of Freiburg, Germany | Freiburg, Germany | Member | |
| Bing | He | | PhD | University of Michigan | Ann Arbor, MI, United States of America | Member | |
| Darren W | Henderson | | | University of Kentucky | Lexington, KY, United States of America | Member | |
| Martin | Hilka | | | APHP Greater Paris University Hospital | Paris, France | Member | |
| John H | Holmes | | MS, PhD | University of Pennsylvania Perelman School of Medicine (inst), Philadelphia, Pennsylvania, United States of America | Philadelphia, PA, United States of America | Member | |
| Petar | Horki | | PhD | Faculty of Medicine and Medical Center, University of Freiburg, Germany | Freiburg, Germany | Member | |
| Kenneth M | Huling | | HS | Harvard Medical School | Boston, MA, United States of America | Member | |
| Anne Sophie | Jannot | | | HEGP, APHP Greater Paris University Hospital | Paris, France | Member | |
| Vianney | Jouhet | | MD,PhD | Bordeaux University Hospital | Bordeaux, France | Member | |
| Ramakanth | Kavuluru | | PhD | University of Kentucky | Lexington, KY, United States of America | Member | |
| Katie | Kirchoff | | MSHI | Medical University of South Carolina | Charleston, South Carolina, United States of America | Member | |
| Ian D | Krantz | | | The Children's Hospital of Philadelphia and the Perelman School of Medicine at the University of Pennsylvania | Philadelphia, PA, United States of America | Member | |
| Detlef | Kraska | | Dr. | University Hospital Erlangen | Erlangen, Germany | Member | |
| Ashok K | Krishnamurthy | | PhD | University of North Carolina, Chapel Hill | Chapel Hill, NC, United States of America | Member | |
| Sehi | L'Yi | | PhD | Harvard Medical School | Boston, MA, United States of America | Member | |
| Trang T | Le | | PhD | University of Pennsylvania Perelman School of Medicine | Philadelphia, PA, United States of America | Member | |
| Judith | Leblanc | | | APHP Greater Paris University Hospital | Paris, France | Member | |
| Andressa RR | Leite | | | Clinical Research Unit of Botucatu Medical School, São Paulo State University, Botucatu, Brazil | Botucatu, Brazil | Member | |
| Guillaume | Lemaitre | | | Université Paris-Saclay, Inria, CEA | Palaiseau, France | Member | |

*Indicates required information. Only first name, last name, and suffix will appear in PubMed.

| ***First Name and Middle Initial(s)** | ***Last Name** | ***Suffix (eg, Jr, III)** | Academic Degrees | Institution | Location (city, state/province, country) | Role or Contribution, eg, chair, principal investigator | Group (if more than 1 Group listed in the byline) and/or Subgroup (eg, Steering Committee) |
|---|---|---|---|---|---|---|---|
| Leslie | Lenert | | MD, MS | Medical University of South Carolina | Charleston, South Carolina, United States of America | Member | |
| Damien | Leprovost | | | Clevy.io | Paris, France | Member | |
| Ne Hooi Will | Loh | | MBBS | National University Health System, Singapore | Lower Kent Ridge Road, Singapore 119074 | Member | |
| Kristine E | Lynch | | PhD | VA Salt Lake City Health Care System | Salt Lake CIty, UT, United States of America | Member | |
| Sadiqa | Mahmood | | | Health Catalyst, INC. | Cambridge, MA, United States of America | Member | |
| Sarah | Maidlow | | | University of Michigan | Ann Arbor, MI, United States of America | Member | |
| Alberto | Malovini | | PhD | Istituti Clinici Scientifici Maugeri SpA SB IRCCS, Pavia, Italy. | Pavia, Italy | Member | |
| Anupama | Maram | | MS | Harvard Medical School | Boston, MA, United States of America | Member | |
| Patricia | Martel | | | APHP Greater Paris University Hospital | Boulogne-Billancourt, France | Member | |
| Aaron J | Masino | | PhD | Children's Hospital of Philadelphia | Philadelphia, PA, United States of America | Member | |
| Michael E | Matheny | | MD | Tennessee Valley Healthcare System Veterans Affairs Medical Center | Nashville, Tennessee, United States of America | Member | |
| Thomas | Maulhardt | | | University Medical Center Freiburg, Germany | Freiburg, Germany | Member | |
| Maria | Mazzitelli | | PhD | University Magna Graecia of Catanzaro, Italy | Catanzaro, ITALY | Member | |
| Michael T | McDuffie | | | Harvard Medical School | Boston, MA, United States of America | Member | |
| Arthur | Mensch | | | ENS, PSL University | Paris, France | Member | |
| Marianna | Milano | | PhD | University Magna Graecia of Catanzaro, Italy | Catanzaro, ITALY | Member | |
| Marcos F | Minicucci | | | Internal Medicine Department of Botucatu Medical School, São Paulo State University, Botucatu, Brazil | Botucatu, Brazil | Member | |
| Jason H | Moore | | PhD | University of Pennsylvania Perelman School of Medicine | Philadelphia, PA, United States of America | Member | |
| Cinta | Moraleda | | MD,PhD | Hospital Universitario 12 de Octubre, Madrid, Spain | Madrid, Spain | Member | |
| Jeffrey S | Morris | | | University of Pennsylvania Perelman School of Medicine | Berwyn, PA, United States of America | Member | |

*Indicates required information. Only first name, last name, and suffix will appear in PubMed.

| *First Name and Middle Initial(s) | *Last Name | *Suffix (eg, Jr, III) | Academic Degrees | Institution | Location (city, state/province, country) | Role or Contribution, eg, chair, principal investigator | Group (if more than 1 Group listed in the byline) and/or Subgroup (eg, Steering Committee) |
|---|---|---|---|---|---|---|---|
| Michele | Morris | | BA | University of Pittsburgh | Pittsburgh, PA, United States of America | Member | |
| Sajad | Mousavi | | PhD | Harvard Medical School | Boston, MA, United States of America | Member | |
| Danielle L | Mowery | | PhD | University of Pennsylvania Perelman School of Medicine | Philadelphia, PA, United States of America | Member | |
| Douglas A | Murad | | | David Geffen School of Medicine at UCLA | Los Angeles, CA, United States of America | Member | |
| Thomas P | Naughton | | BA | Harvard Medical School | Boston, MA, United States of America | Member | |
| James B | Norman | | | Harvard Medical School | Boston, MA, United States of America | Member | |
| Jihad | Obeid | | MD, FAMIA | Medical University of South Carolina | Charleston, South Carolina, United States of America | Member | |
| Marina P | Okoshi | | PhD | Internal Medicine Department of Botucatu Medical School, São Paulo State University, Botucatu, Brazil | Botucatu, Brazil | Member | |
| Karen L | Olson | | PhD | Boston Children's Hospital and Harvard Medical School | Boston, MA, United States of America | Member | |
| Gilbert S | Omenn | | MD, PhD | University of Michigan | Ann Arbor, MI, United States of America | Member | |
| Nina | Orlova | | | APHP Greater Paris University Hospital | Paris, France | Member | |
| Brian D | Ostasiewski | | BS | Wake Forest School of Medicine | Winston-Salem, NC, United States of America | Member | |
| Nathan P | Palmer | | PhD | Harvard Medical School | Boston, MA, United States of America | Member | |
| Nicolas | Paris | | | APHP Greater Paris University Hospital | Paris, France | Member | |
| Emily R | Pfaff | | PhD | UNC Chapel Hill | Chapel Hill, NC, United States of America | Member | |
| Danielle | Pillion | | MS | Harvard Medical School | Boston, MA, United States of America | Member | |
| Hans U | Prokosch | | | University of Erlangen-Nürnberg | Erlangen, Germany | Member | |
| Robson A | Prudente | | PhD | Clinical Research Unit São Paulo State University, Brazil | Botucatu, Brazil | Member | |
| Víctor | Quirós González | | MS | Hospital Universitario 12 de Octubre, Madrid, Spain | Madrid, Spain | Member | |
| Rachel B | Ramoni | | DMD, ScD | Department of Veterans Affairs | Washington, DC, United States of America | Member | |
| Maryna | Raskin | | | Health Catalyst, INC. | Cambridge, MA, United States of America | Member | |
| Siegbert | Rieg | | MD | Medical Center – University of Freiburg, Faculty of Medicine | Freiburg, Germany | Member | |

*Indicates required information. Only first name, last name, and suffix will appear in PubMed.

| *First Name and Middle Initial(s) | *Last Name | *Suffix (eg, Jr, III) | Academic Degrees | Institution | Location (city, state/province, country) | Role or Contribution, eg, chair, principal investigator | Group (if more than 1 Group listed in the byline) and/or Subgroup (eg, Steering Committee) |
|---|---|---|---|---|---|---|---|
| Gustavo | Roig Domínguez | | MS | Hospital Universitario 12 de Octubre, Madrid, Spain | Madrid, Spain | Member | |
| Pablo | Rojo | | MD,PhD | Hospital Universitario 12 de Octubre, Madrid, Spain | Madrid, Spain | Member | |
| Carlos | Sáez | | PhD | Universitat Politècnica de València, Spain | Valencia, Spain | Member | |
| Elisa | Salamanca | | | APHP Greater Paris University Hospital | Paris, France | Member | |
| Malarkodi J | Samayamuthu | | MD | University of Pittsburgh | Pittsburgh, PA, United States of America | Member | |
| Arnaud | Sandrin | | | APHP Greater Paris University Hospital | Paris, France | Member | |
| Janaina CC | Santos | | MS | Clinical Research Unit of Botucatu Medical School, São Paulo State University, Botucatu, Brazil | Botucatu, Brazil | Member | |
| Maria | Savino | | MS | Management Engineer, Direction | Pavia PV, Italy | Member | |
| Emily R | Schriver | | MS | University of Pennsylvania Health System | Philadelphia, PA, United States of America | Member | |
| Juergen | Schuettler | | | University Hospital Erlangen, FAU Erlangen-Nürnberg, Germany | Erlangen, Germany | Member | |
| Luigia | Scudeller | | MD, MSc | IRCCS Ca' Granda Ospedale Maggiore Policlinico di Milano | Milan, Italy | Member | |
| Patricia | Serre | | | APHP Greater Paris University Hospital | Paris, France | Member | |
| Domenick | Silvio | | | University of Michigan | Ann Arbor, MI, United States of America | Member | |
| Piotr | Sliz | | | Boston Children's Hospital | Boston, MA, United States of America | Member | |
| Jiyeon | Son | | MD | University of Pittsburgh Medical Center | Pittsburgh PA, United States of America | Member | |
| Charles | Sonday | | | St. Luke's University Health Network, Bethlehm PA | Bethlehem, PA, United States of America | Member | |
| Bryce WQ | Tan | | MBBS | National University Hospital, Singapore | 1E Kent Ridge Road, Singapore 119228 | Member | |
| Byorn WL | Tan | | MBBS | National University Hospital, Singapore | 1E Kent Ridge Road, Singapore 119228 | Member | |
| Suzana E | Tanni | | PhD | Internal Medicine Department of Botucatu Medical School, São Paulo State University, Botucatu, Brazil | Botucatu, Brazil | Member | |

Supplemental Online Content: Nonauthor Collaborators

*Indicates required information. Only first name, last name, and suffix will appear in PubMed.

| *First Name and Middle Initial(s) | *Last Name | *Suffix (eg, Jr, III) | Academic Degrees | Institution | Location (city, state/province, country) | Role or Contribution, eg, chair, principal investigator | Group (if more than 1 Group listed in the byline) and/or Subgroup (eg, Steering Committee) |
|---|---|---|---|---|---|---|---|
| Ana I | Terriza Torres | | MS | Hospital Universitario 12 de Octubre, Madrid, Spain | Madrid, Spain | Member | |
| Valentina | Tibollo | | MS | Istituti Clinici Scientifici Maugeri SpA SB IRCCS, Pavia, Italy. | Pavia, Italy | Member | |
| Carlo | Torti | | PhD | University Magna Graecia of Catanzaro, Italy | Catanzaro, ITALY | Member | |
| Enrico M | Trecarichi | | PhD | University Magna Graecia of Catanzaro, Italy | Catanzaro, ITALY | Member | |
| Yi-Ju | Tseng | | PhD | Chang Gung University | Guishan, Taoyuan, Taiwan | Member | |
| Andrew K | Vallejos | | | Clinical & Translational Science Institute, Medical College of Wisconsin | Milwaukee, WI, United States of America | Member | |
| Gael | Varoquaux | | | Université Paris-Saclay, Inria, CEA, Montréal Neurological Institute, McGill University | Palaiseau, France | Member | |
| Jill-Jênn | Vie | | | SequeL, Inria Lille | Villeneuve-d'Ascq, France | Member | |
| Michele | Vitacca | | MD, PhD | ICS S. Maugeri IRCCS Pavia Italy | Lumezzane (Bs), Italy | Member | |
| Kavishwar B | Wagholikar | | MBBS, PhD | Department of Medicine, Massachusetts General Hospital, Boston, USA | Boston MA, United States of America | Member | |
| Lemuel R | Waitman | | | University of Missouri, Columbia. MO | Columbia, MO, United States of America | Member | |
| Demian | Wassermann | | | Université Paris-Saclay, Inria, CEA | Palaiseau, France | Member | |
| Yuan | William | | | Harvard Medical School | Boston, MA, United States of America | Member | |
| Zongqi | Xia | | MD, PhD | University of Pittsburgh | Pittsburgh, PA, United States of America | Member | |
| Nadir | Yehya | | MD, MSCE | Children's Hospital of Philadelphia and University of Pennsylvania | Philadelphia, PA, United States of America | Member | |
| Alberto | Zambelli | | | ASST Papa Giovanni XXIII, Bergamo | Bergamo, Italy | Member | |
| Harrison G | Zhang | | | Harvard Medical School | Boston, MA, United States of America | Member | |
| Chiara | Zucco | | PhD | University Magna Graecia of Catanzaro, Italy | Catanzaro, ITALY | Member | |
